# High expression of integrin-binding sialoprotein (IBSP) is associated with poor prognosis of osteosarcoma

**DOI:** 10.18632/aging.205235

**Published:** 2023-11-22

**Authors:** Yihang Ma, Bing Chen, Boyin Zhang, Chao Zhang, Qingsan Zhu, Xu Wang, Zhengang Liu, Haochuan Liu

**Affiliations:** 1 Department of Spine Surgery, China-Japan Union Hospital of Jilin University, Changchun 130033, People’s Republic of China; 2 Department of Anesthesiology, China-Japan Union Hospital of Jilin University, Changchun 130033, People’s Republic of China; 3 Department of Operating Room, China-Japan Union Hospital of Jilin University, Changchun 130033, People’s Republic of China

**Keywords:** IBSP, overall survival, disease specific survival, osteosarcoma, biomarker

## Abstract

Introduction: Osteosarcoma is a malignant tumor, accounting for 20% of primary malignant bone tumors worldwide. However, the role of IBSP as a biomarker in osteosarcoma progression has not been studied yet.

Methods: 85 cases of IBSP expression and clinical characteristics were obtained from TARGET database. Through the Kaplan-Meier curve, subgroup analysis, and univariate and multivariate Cox analysis, we further assessed the independent predictive capacity of IBSP expression for overall survival (OS) and relapse-free survival (RFS).

Results: The mRNA expression of IBSP was higher in osteosarcoma than normal tissue (*P* < 0.0001). IBSP expression grouped by vital status showed statistical differences (*P* = 0.042). The race (*P* = 0.0183), vital status (*P* = 0.0034), and sample type (*P* = 0.0020) showed significant differences. IBSP expression exhibited satisfied diagnostic ability for osteosarcoma. The univariate and multivariate analysis confirmed that IBSP expression was an independent risk factor for OS (HR = 3.425, 95% CI: 1.604–7.313, *P* = 0.002) and RFS (HR = 3.377, 95% CI: 1.775–6.424, *P* < 0.001) in osteosarcoma patients. High IBSP expression was significantly associated with poor OS and RFS (*P* < 0.0001). The higher IBSP expression was observed in osteosarcoma (*P* < 0.001), confirmed by the IHC staining. The CCK-8 and colony formation assay showed that IBSP knockdown inhibits cell proliferation while overexpression promotes cell proliferation (*P* < 0.05).

Conclusion: High expression of IBSP was associated with poor OS and RFS. IBSP could serve as a potential biomarker for osteosarcoma, which could aid in early detection and disease monitoring.

## INTRODUCTION

Osteosarcoma is a malignant tumor derived from mesenchymal tissue, which is composed of spindle shaped stromal cells that produce osteoid tissue [1]. Osteosarcoma accounts for 20% of primary malignant bone tumors worldwide [2]. Studies have shown that osteosarcoma mostly occurs in juvenile patients [3]. Osteosarcomas often occur in the proximal tibia, proximal humerus, and distal femur, and less commonly in regions such as the sacrum, pelvis, and spine [4]. More relevant studies showed that the vast majority of patients with osteosarcoma have only one lesion. Osteosarcoma, with local swelling and pain as the main symptoms, can be accompanied by joint dysfunction, and very few patients can have pathological fractures [5]. Painful symptoms of osteosarcoma are not easily identified and are not of low malignancy [3]. Recently, biomarkers have been identified that are associated with osteosarcoma progression and could serve as potential therapeutic targets [6].

IBSP (Integrin-binding sialoprotein), also known as Bone sialoprotein 2 (BSP2), is a glycoprotein that is mainly expressed in bone tissue [7]. High levels of IBSP have been found in various types of cancer, including colorectal cancer breast, esophageal squamous cell carcinoma [8, 9]. Studies have shown that IBSP plays a significant role in tumor growth, invasion, and metastasis by promoting angiogenesis, cell migration and adhesion, and immune microenvironment [10, 11]. Several studies have suggested that IBSP may serve as a potential biomarker for cancer diagnosis and prognosis. For instance, a study conducted in breast cancer patients found that IBSP could induce osteolytic bone metastasis of estrogen receptor-positive breast cancer [7]. However, the function of IBSP in osteosarcoma has not been studied yet.

In this bioinformatic analysis of osteosarcoma, we obtained the data of IBSP expression and clinical characteristics from the publicly available cancer genome database TARGET and examined the expression levels of IBSP in tumor samples. Diagnostic ability of IBSP for osteosarcoma was studied. The overall survival and relapse free survival in osteosarcoma patients were analyzed. Finally, our bioinformatic analysis of osteosarcoma revealed that IBSP expression is significantly higher in tumor tissues compared to normal bone tissues. Furthermore, higher IBSP expression is significantly correlated with poor overall and relapse-free survival in osteosarcoma patients. These findings suggest that IBSP could serve as a prognostic biomarker for predicting outcomes in osteosarcoma patients, as well as aid in early detection and monitoring of the disease.

## MATERIALS AND METHODS

### Data mining

The data of IBSP expression and clinical characteristics were obtained from Therapeutically Applicable Research to Generate Effective Treatments (TARGET) database (https://ocg.cancer.gov/programs/target) [12]. The mRNA expression profile was analyzed using algorithm log2 (x + 1).

### Patients and samples

The osteosarcoma and normal bone tissues for validation of IBSP expression were collected from 15 patients admitted to the department of spine surgery, China-Japan Union Hospital of Jilin University from June 2022 to December 2022. The tissue type was determined by the pathologist.

The osteosarcoma and normal bone tissues for another cohort consisting of 21 living patients and 11 deceased patients were collected from 32 patients admitted to the department of spine surgery, China-Japan Union Hospital of Jilin University from January 2020 to December 2022. The tissue type was determined by the pathologist. The patients characteristics were shown in Supplementary Table 1.

### Comparison analysis

The non-parametric rank sum test, Wilcoxon rank sum test, and Kruskal-Wall test were used for assessing IBSP expression, comparing differences between both groups, comparing differences among multiple groups, respectively.

### Receiver operating characteristic curves

The receiver operating characteristic (ROC) curves were plotted to evaluate the diagnostic capability of IBSP expression through pROC program [13]. The area under the curve (AUC) was then calculated and the cutoff value was obtained for grouping of high- or low-IBSP expression [14].

### Kaplan-Meier curves

The overall survival and relapse free survival in osteosarcoma patients were analyzed by Kaplan-Meier curve [15]. The subgroup analysis including age, gender, site, and surgery was performed.

### Real-time quantitative PCR

Total RNA was extracted using TRIzol (Invitrogen, USA). 1 mL of isolated RNA was used for the reverse transcription. The real-time quantitative PCR (RT-qPCR) procedure was then completed. The primers are as follows: IBSP (Forward: 5′-GCATGCCTACTTTTATCCTCATTTAA-3′; Reverse: 5′-TCTTCTGAACTGTCATCTCCATTTTC-3′), and β-actin (Forward: 5′-GGAGCGAGATCCCTCCAAAAT-3′; Reverse: 5′-GGCTGTTGTCATACTTCTCATGG-3′).

### Immunohistochemistry

After pre-treatments, the tissue sections were probed with primary antibodies against IBSP (HY-P80969, MedChemExpress, USA) and secondary antibody (3900S, Cell Signaling Technology, USA) using the Immunohistochemistry Application Solutions Kit (#13079, Cell Signaling Technology, USA) according to the manufacturer’s instructions. The immunohistochemistry staining was performed as reported [16].

### Cell culture and transfection

The normal (HaCaT and HOB) and cancer (SaOS2, U2-OS, and MG-63) cell lines were grown in corresponding medium, which contains 10% fetal bovine serum and 1% penicillin-streptomycin solution, at 37°C in a 5% CO_2_ humidified incubator. The si-IBSP and O-IBSP plasmids were then transfected into the cell for knockdown and overexpression, respectively.

### Cell proliferation assay

CCK-8 and colony formation assays were performed to evaluate the cell proliferation. The cells treated with si-NC, si-IBSP and O-IBSP were cultured for 48 h. The absorbance at 450 nm was then measured after adding 10 μl of CCK-8 reagent. The colony formation assay was further performed as reported [16].

### Statistical analysis

The statistical analysis was carried out through R3.5.1 [17]. The chi-square test and Fisher’s exact test were used to evaluate the association between IBSP expression and clinical characteristics. The univariate and multivariate Cox analysis was used to evaluate the predictive capability of IBSP expression. *P* < 0.05 was statistically significant.

## RESULTS

### Characteristics of patients with osteosarcoma

First, we analyzed the data from database and summarized the characteristics of 85 patients with osteosarcoma (Table 1). There were 40 (47.06%) patients less than 14 years old. Male patients (55.29%) were more than female patients (44.71%). As for race, most patients (61.18%) were white. All of the patients had metastasis and 74.12% patients had progression.

**Table 1:** Characteristics of patients with osteosarcoma.

	**Characteristics**	**Numbers of cases (%)**
Age	<14	40 (47.06)
	>14	45 (52.94)
Gender	Female	38 (44.71)
	Male	47 (55.29)
Race	UA	10 (11.76)
	Asian	6 (7.06)
	Black or African American	7 (8.24)
	Unknown	10 (11.76)
	White	52 (61.18)
Metastasis	Metastasis	85 (100)
Site	UA	31 (36.47)
	Distal	28 (32.94)
	Other	3 (3.53)
	Proximal	23 (27.06)
Surgery	UA	39 (45.88)
	Amputation	5 (5.88)
	Limb sparing	41 (48.24)
Progression	No	22 (25.88)
	Yes	63 (74.12)
Vital status	Alive	58 (68.24)
	Dead	27 (31.76)
Sample type	UA	1 (1.18)
	Censored	11 (12.94)
	Death	2 (2.35)
	No	32 (37.65)
	Relapse	38 (44.71)
	SMN	1 (1.18)
IBSP	High	22 (25.88)
	Low	63 (74.12)

Abbreviation: UA: unavailable.

### High expression of IBSP in osteosarcoma

**Figure 1:**
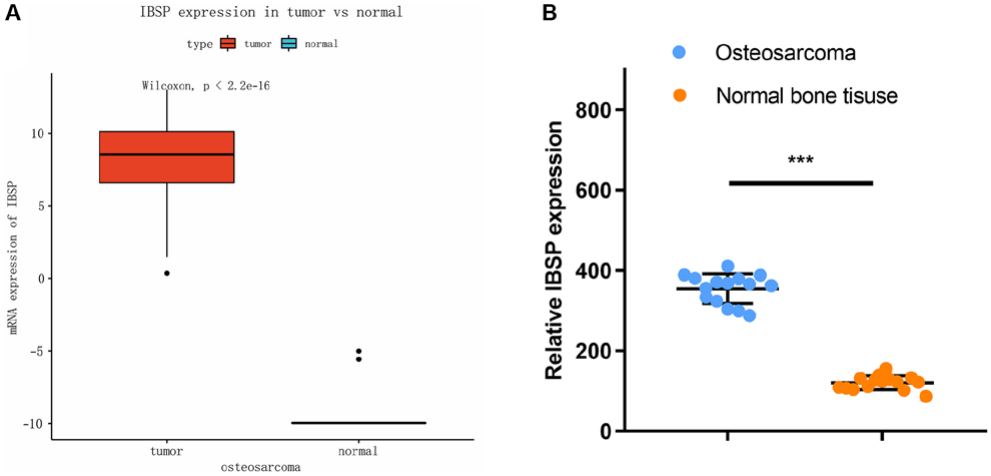
**Expression of IBSP in osteosarcoma and normal tissue.** (**A**) TARGET database analysis. (**B**) Validation in our collected 15 osteosarcoma and normal bone tissues. ^***^P < 0.001.

As shown in Figure 1A, the mRNA expression of IBSP was higher in osteosarcoma than normal tissue with *P*-value less than 0.0001. Moreover, we validated the results in our collected 15 osteosarcoma and normal bone tissues (Figure 1B). The osteosarcoma and normal bone tissues were collected from 15 patients admitted to the department of spine surgery, China-Japan Union Hospital of Jilin University from June 2022 to December 2022. The tissue type was determined by the pathologist.

**Figure 2:**
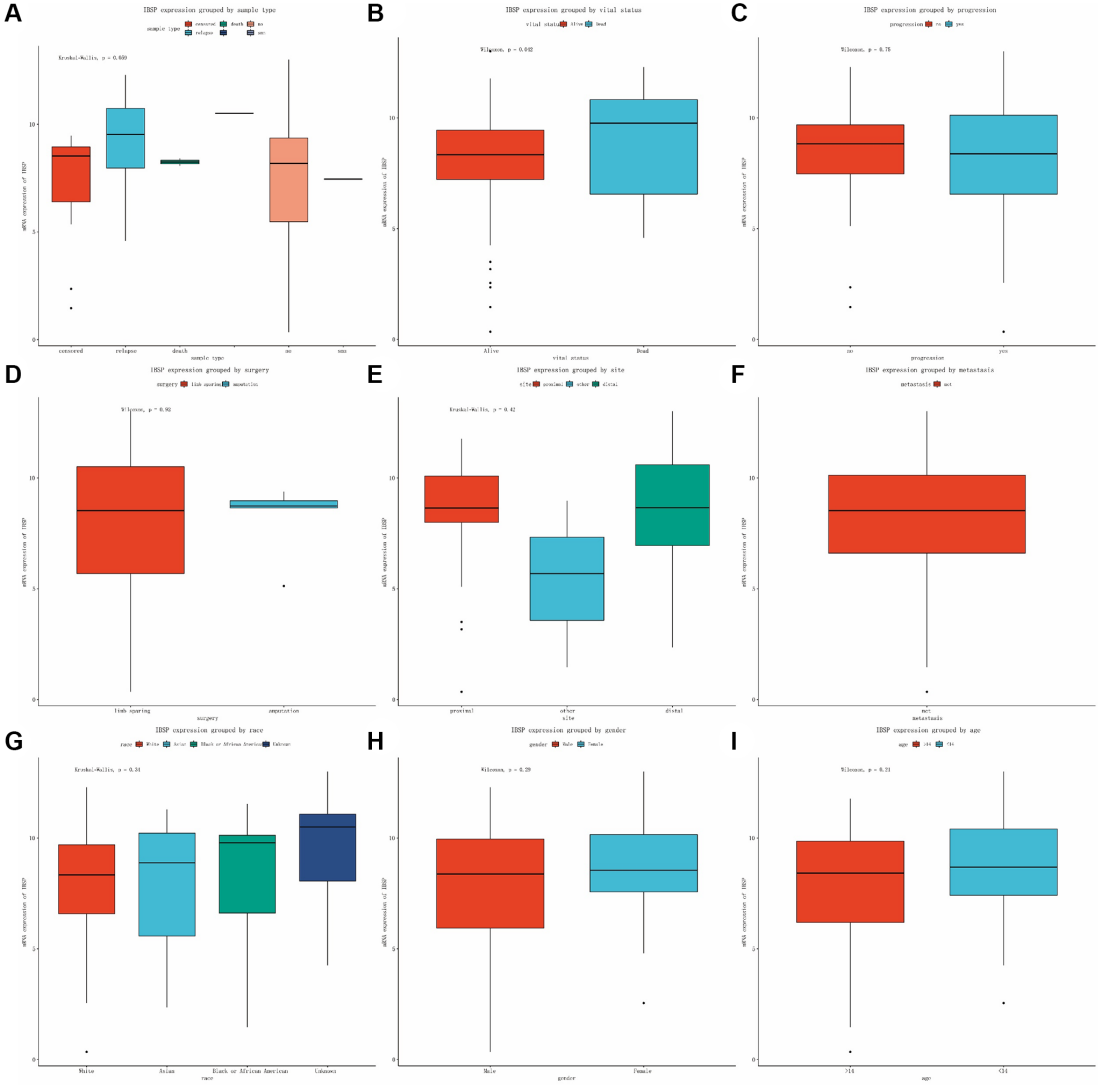
**Expression of IBSP in subgroups.** (**A**) Sample type. (**B**) Vital status. (**C**) Progression. (**D**) Surgery. (**E**) Site. (**F**) Metastasis. (**G**) Race. (**H**) Gender. (**I**) Age.

This encouraged us to further evaluate the IBSP expression in subgroups (Figure 2A–2I). Only IBSP expression grouped by vital status showed statistical differences (*P* = 0.042). However, sample type, progression, surgery, site, metastasis, race, gender, and age showed no significances (*P* > 0.05).

### The association between IBSP expression and clinical characteristics

Then, we performed the logistic analysis of the association between IBSP expression and clinical characteristics (Table 2). The race (*P* = 0.0183), vital status (*P* = 0.0034), and sample type (*P* = 0.0020) showed significant differences, while other parameters were not statistically significant.

**Table 2:** Logistic analysis of the association between IBSP expression and clinical characteristics.

**Parameter**		***N***	**High**	**Percentage**	**Low**	**Percentage**	**χ2**	***P***
Age	<14	40	13	(59.09)	27	(42.86)	1.1348	0.2868
	>14	45	9	(40.91)	36	(57.14)		
Gender	Female	38	12	(54.55)	26	(41.27)	0.6875	0.4070
	Male	47	10	(45.45)	37	(58.73)		
Race	Asian	6	2	(10)	4	(7.27)	9.0856	**0.0183**
	Black or African American	7	3	(15)	4	(7.27)		
	Unknown	10	6	(30)	4	(7.27)		
	White	52	9	(45)	43	(78.18)		
Site	Distal	28	9	(60)	19	(48.72)	1.4526	0.7026
	Other	3	0	(0)	3	(7.69)		
	Proximal	23	6	(40)	17	(43.59)		
Surgery	Amputation	5	0	(0)	5	(15.15)	0.9227	0.3368
	Limb sparing	41	13	(100)	28	(84.85)		
Progression	No	22	5	(22.73)	17	(26.98)	0.012	0.9126
	Yes	63	17	(77.27)	46	(73.02)		
Vital status	Alive	58	9	(40.91)	49	(77.78)	8.5957	**0.0034**
	Dead	27	13	(59.09)	14	(22.22)		
Sample type	Censored	11	0	(0)	11	(17.46)	15.2281	**0.0020**
	Death	2	0	(0)	2	(3.17)		
	No	32	4	(19.05)	28	(44.44)		
	Relapse	38	17	(80.95)	21	(33.33)		
	SMN	1	0	(0)	1	(1.59)		

### Diagnostic ability of IBSP for osteosarcoma

**Figure 3:**
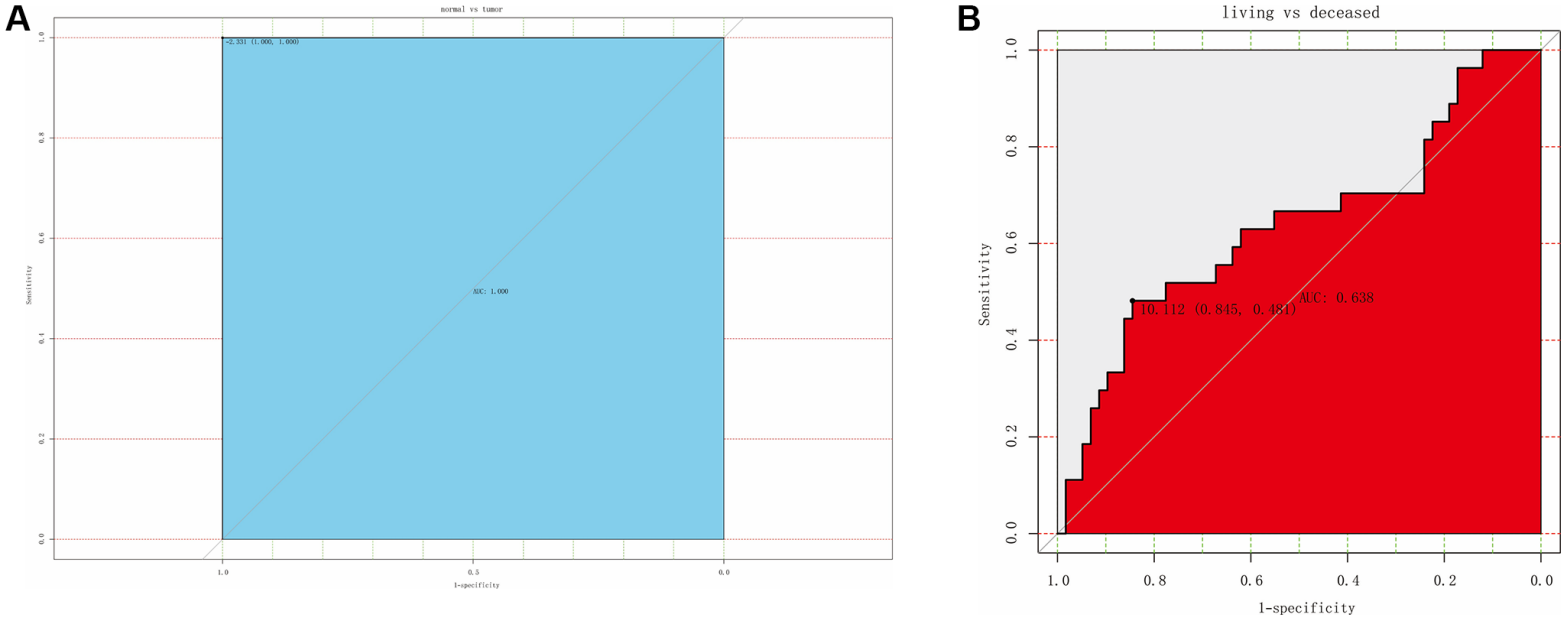
**ROC curve for assessing the diagnostic ability of IBSP expression.** (**A**) The diagnostic value of IBSP in osteosarcoma. (**B**) The diagnostic value of IBSP for living vs. deceased patients.

As shown in Figure 3A, IBSP expression exhibited satisfied diagnostic ability for osteosarcoma with AUC of 1.000 and cut-off value of −2.331. Besides, we further studied the diagnostic ability of IBSP for living vs. deceased patients (Figure 3B). The result was promising with AUC of 0.638 and cut-off value of 10.112.

### Overall survival in osteosarcoma patients

**Figure 4:**
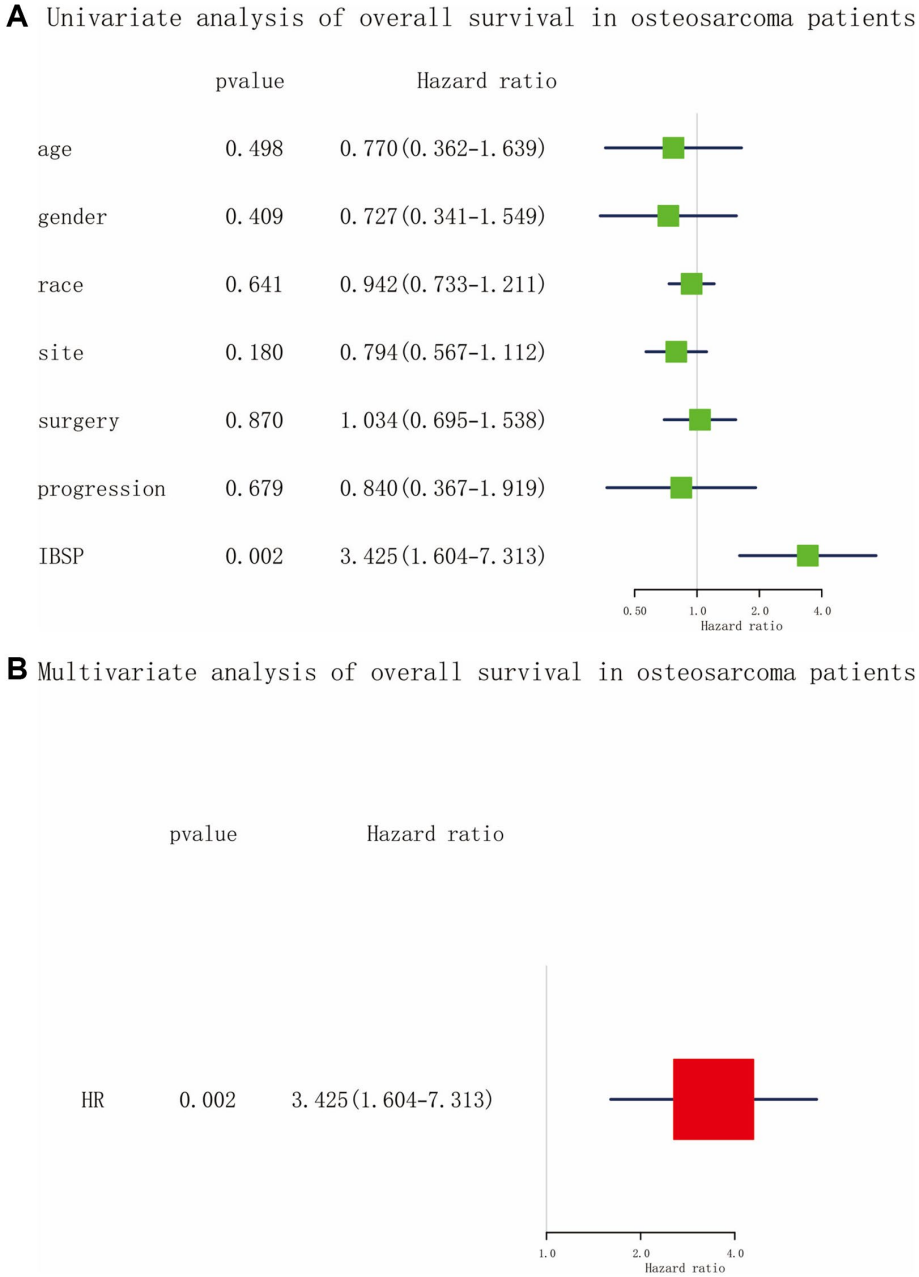
**Cox analysis of overall survival in osteosarcoma.** (**A**) Univariate analysis. (**B**) Multivariate analysis.

**Figure 5:**
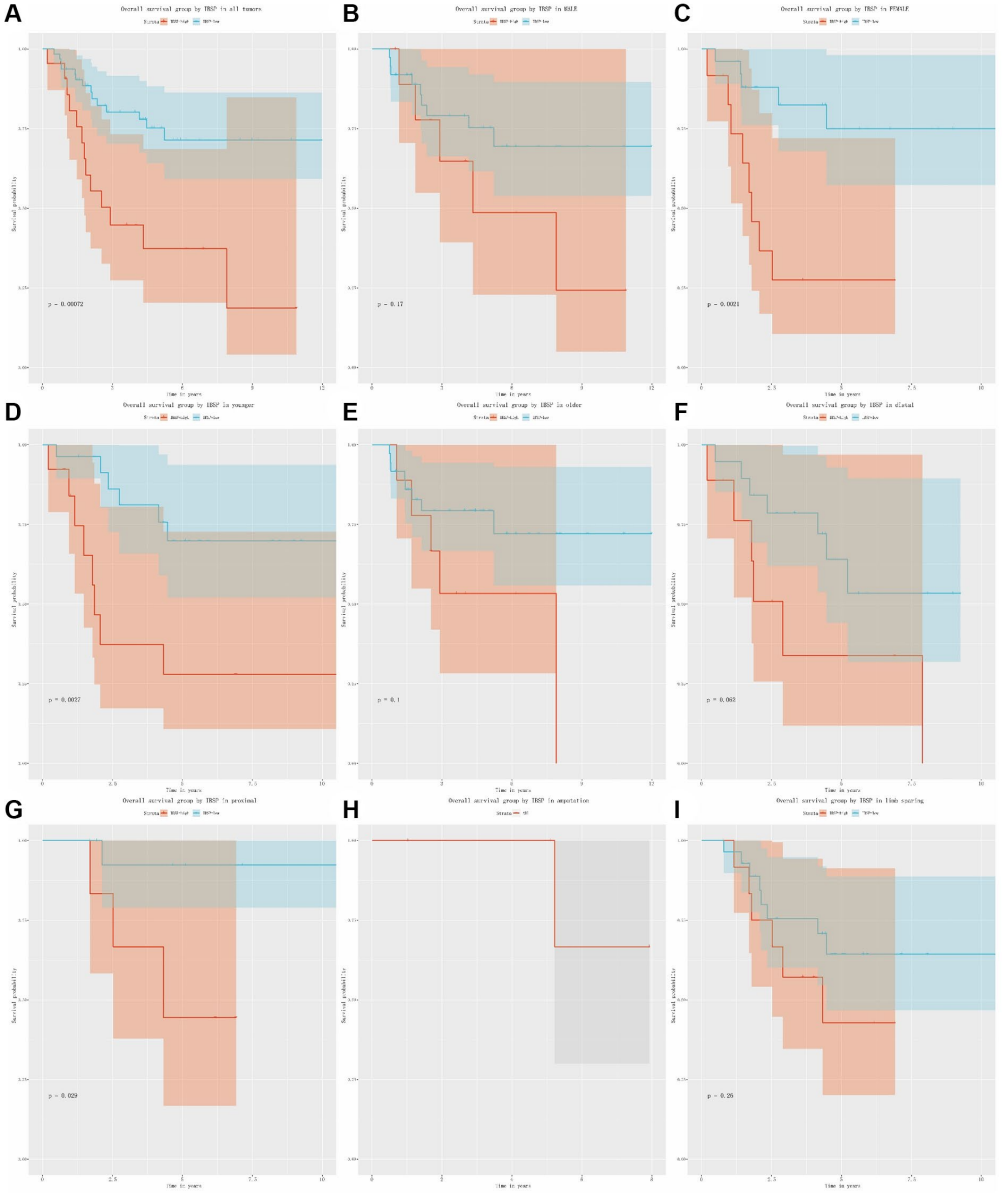
**The relationship between IBSP expression and overall survival.** (**A**) Overall survival group by IBSP in all tumors. (**B**–**I**) Overall survival group by IBSP in male, female, younger, older, distal, proximal, amputation, and limb sparing.

As shown in Figure 4A, 4B, the univariate and multivariate Cox analysis confirmed that IBSP expression was an independent risk factor (HR = 3.425, 95% CI: 1.604–7.313, *P* = 0.002) for overall survival in osteosarcoma patients. Furthermore, we plotted the Kaplan-Meier curves (Figure 5A–5I). High IBSP expression was significantly associated with poor overall survival (*P* < 0.001). As for subgroup analysis, high IBSP expression was significantly associated with poor overall in female patients (*P* = 0.0021), younger patients (*P* = 0.0027), and proximal (*P* = 0.029) patients.

### Relapse free survival in osteosarcoma patients

**Figure 6:**
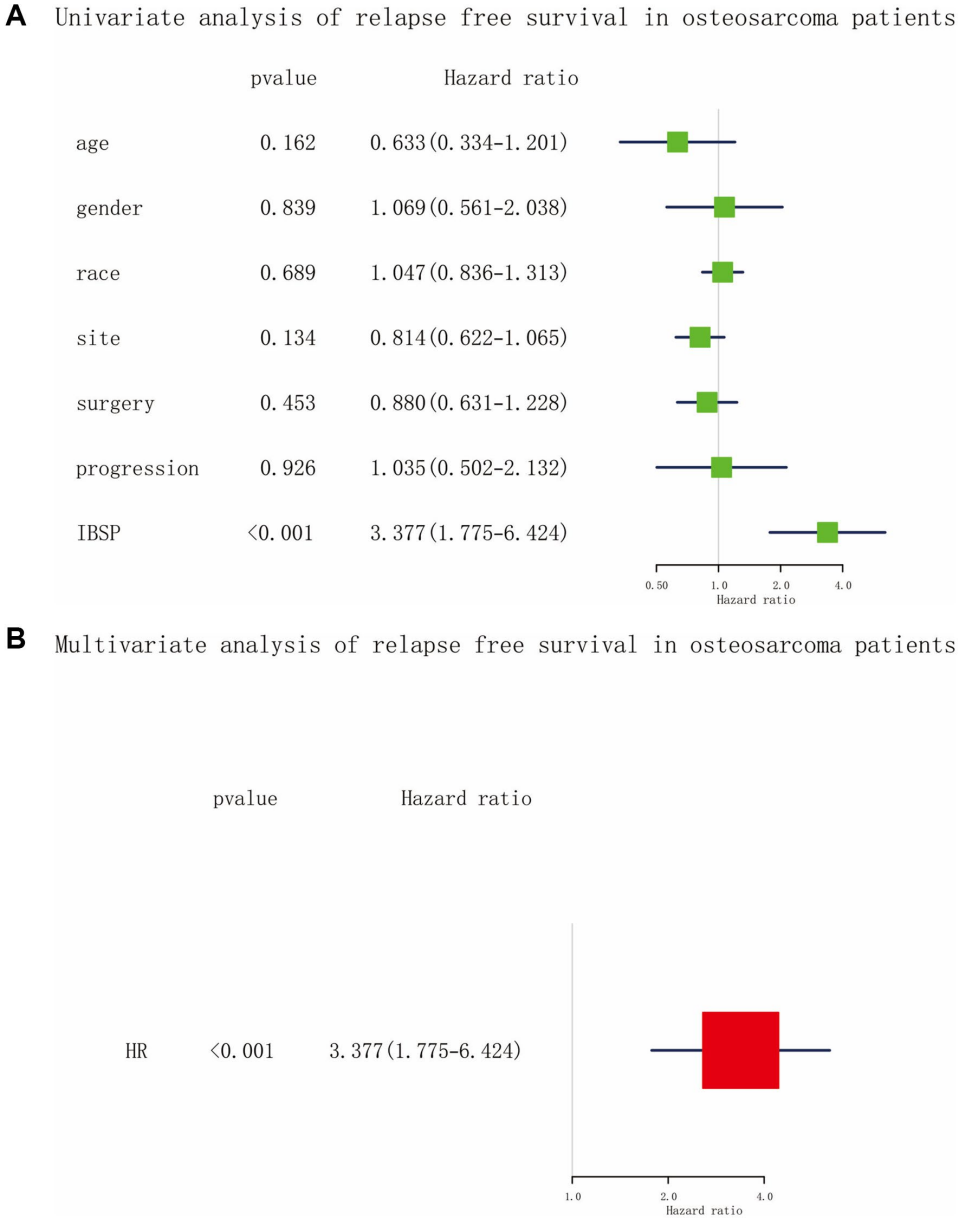
**Cox analysis of relapse free survival in osteosarcoma.** (**A**) Univariate analysis. (**B**) Multivariate analysis.

**Figure 7:**
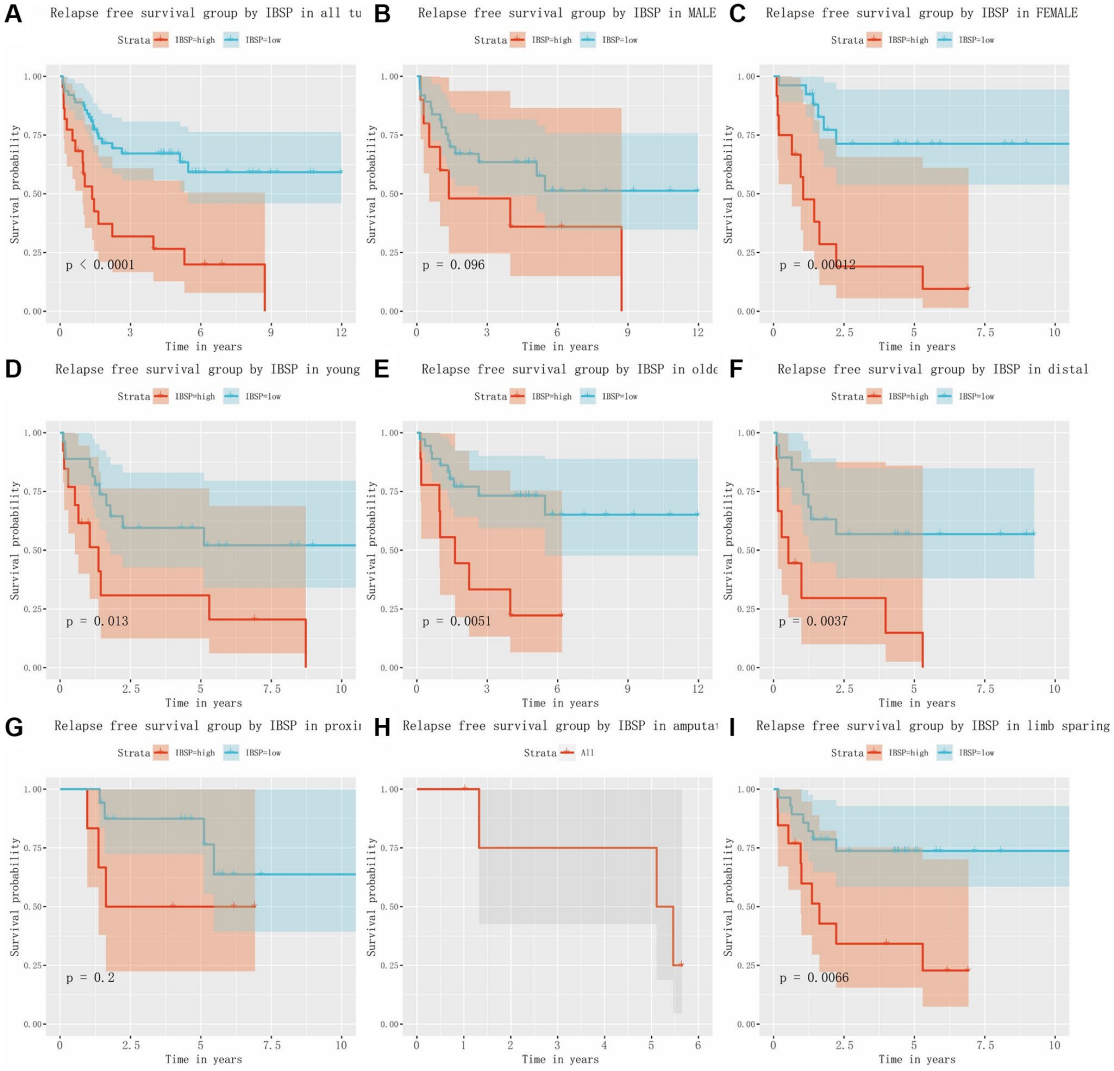
**The relationship between IBSP expression and relapse free survival.** (**A**) Relapse free survival group by IBSP in all tumors. (**B**–**I**) Relapse free survival group by IBSP in male, female, younger, older, distal, proximal, amputation, and limb sparing.

As shown in Figure 6A, 6B, the univariate and multivariate Cox analysis confirmed that IBSP expression was an independent risk factor (HR = 3.377, 95% CI: 1.775-6.424, *P* < 0.001) for relapse free survival in osteosarcoma patients. Furthermore, we plotted the Kaplan-Meier curves (Figure 7A–7I). High IBSP expression was significantly associated with poor relapse free survival (*P* < 0.0001). As for subgroup analysis, high IBSP expression was significantly associated with poor overall in female patients (*P* < 0.001), younger patients (*P* = 0.013), older patients (*P* = 0.0051), distal (*P* = 0.0037), and limb sparing (*P* = 0.0066) patients.

### High IBSP expression is associated with poor survival

**Figure 8:**
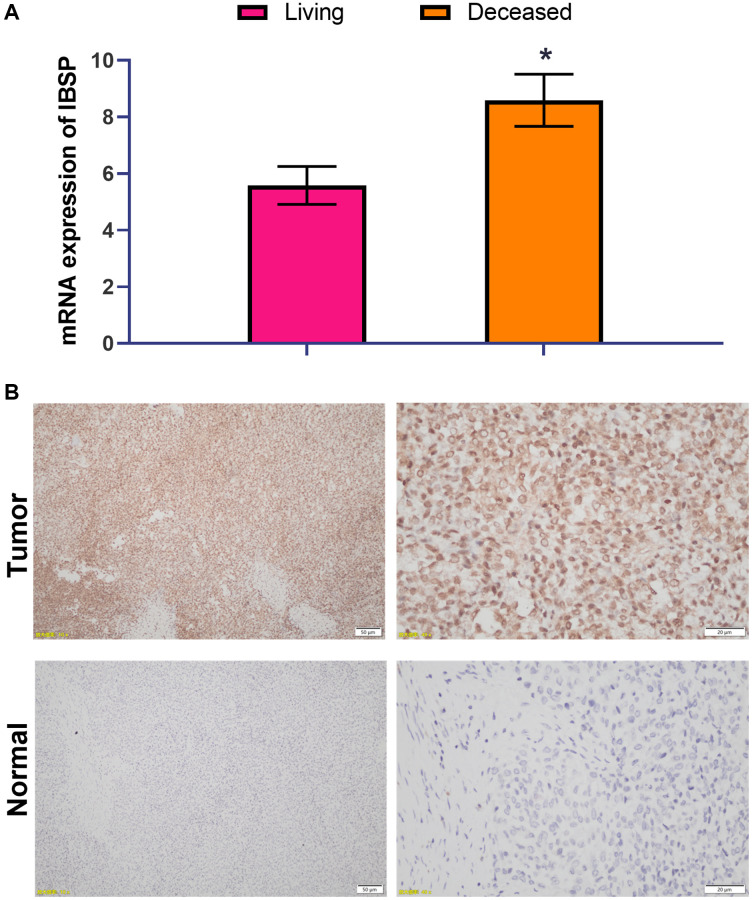
**High IBSP expression is associated with poor survival.** (**A**) The prognostic significance of IBSP expression was validated by RT-PCR with another cohort consisting of 21 living patients and 11 deceased patients. (**B**) The protein expression of IBSP by IHC staining. Scale bar = 50 μm (left panel); scale bar = 20 μm (right panel). ^*^P < 0.05.

The prognostic significance of IBSP expression was validated by RT-PCR with another cohort consisting of 21 living patients and 11 deceased patients (Figure 8A). Higher NPC2 expression was associated with poor survival (*P* < 0.05). We further validated the protein expression of IBSP by IHC staining (Figure 8B). IBSP was highly expressed in tumor tissue compared with normal tissue.

### IBSP knockdown inhibits cell proliferation

**Figure 9:**
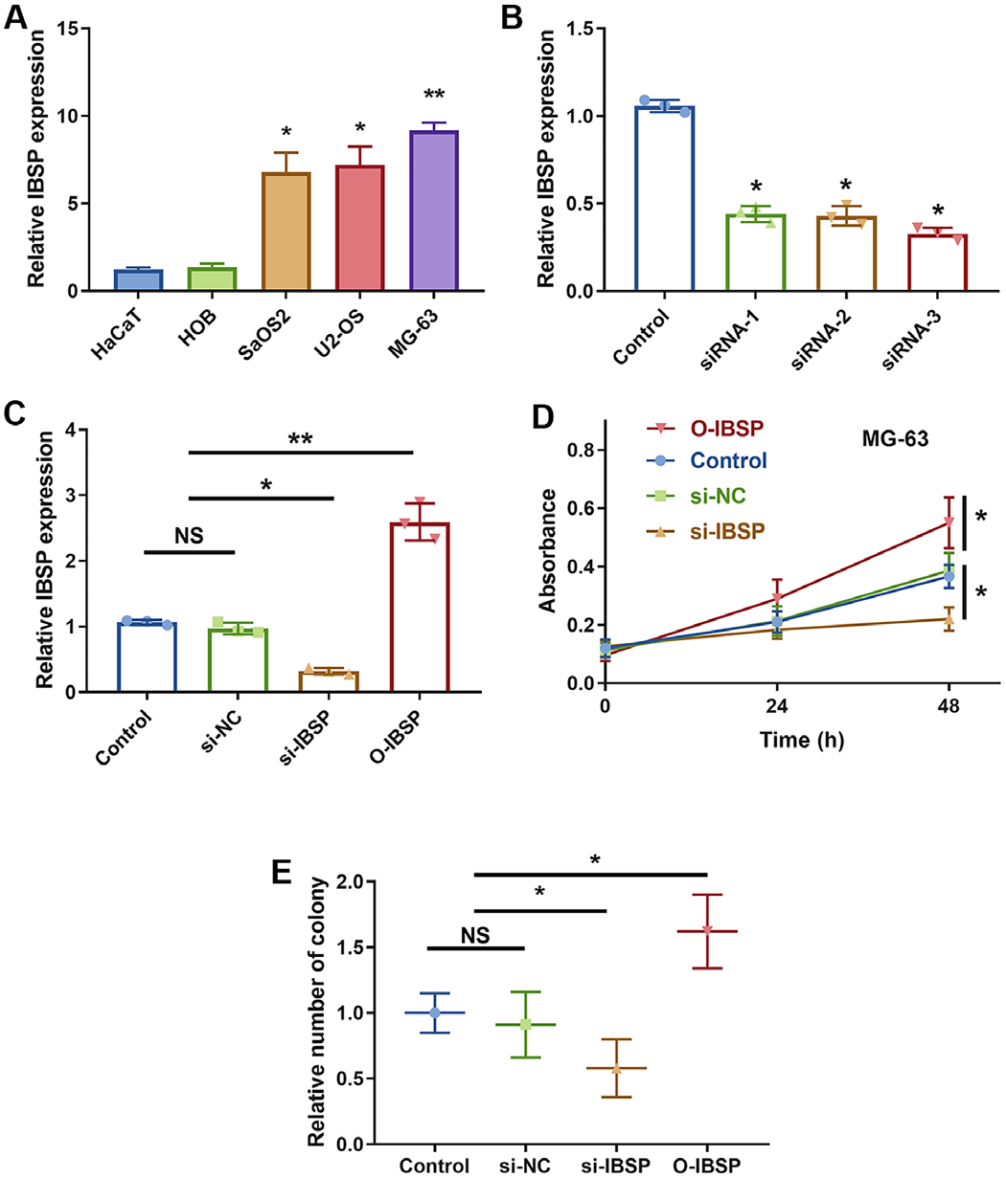
**IBSP knockdown inhibits cell proliferation.** (**A**) The IBSP expression in normal cell lines including HaCaT and HOB, and osteosarcoma cells including SaOS2, U2-OS, and MG-63. (**B**) The knockdown efficiency. (**C**) The overexpression efficiency. (**D**) The CCK-8 assay showing cell proliferation. (**E**) The colony formation assay showing cell proliferation. Abbreviation: NS: no significance. ^*^P < 0.05, ^**^P < 0.01.

The IBSP expression was evaluated in normal cell lines including HaCaT and HOB (Human Osteoblasts), and osteosarcoma cells including SaOS2, U2-OS, and MG-63. The IBSP expression was higher in osteosarcoma cells (*P* < 0.05, Figure 9A). Particularly, the IBSP expression was highest in MG-63, which was used for subsequent cell proliferation assay.

The knockdown and overexpression efficiency were examined and confirmed (Figure 9B, 9C). The siRNA-3 treated cell showed most significant decreased IBSP expression (*P* < 0.05), which was used as si-IBSP. The CCK-8 assay showed that IBSP knockdown inhibits MG-63 cell proliferation while overexpression promotes cell proliferation (*P* < 0.05, Figure 9D). The colony formation assay showed the consistent results (Figure 9E).

## DISCUSSION

Osteosarcoma is a malignant bone tumor that primarily affects children and young adults. Despite advancements in treatment, it remains a challenging disease with high mortality rates [18]. The prognosis for patients with osteosarcoma largely depends on cancer stage, tumor location, and other clinical factors. Osteosarcoma is heterogeneous and causes treatment difficulties, and patient survival has consistently not improved tremendously over the past decade or so [19]. Prognosis related markers among studies in osteosarcoma have been shown to improve the diagnostic and prognostic evaluation of osteosarcoma [20].

Osteosarcoma is a rare but aggressive bone cancer that primarily affects children and teenagers [21]. Early diagnosis and treatment are essential for better prognosis and improved survival rates. Biomarkers, the molecules that can indicate the presence, progression, or response to treatment of a disease, have the potential to enhance the accuracy and speed of osteosarcoma diagnosis and monitoring [22]. One such biomarker is the IBSP, also known as bone sialoprotein II, a non-collagenous glycoprotein that is primarily expressed by bone and cartilage cells [23]. IBSP has been implicated in bone matrix mineralization and turnover, as well as in regulating cell adhesion, proliferation, and differentiation processes [24]. IBSP has also been shown to play a role in tumorigenesis and metastasis in multiple types of cancer, including osteosarcoma [25]. IBSP has been associated with cancer stem cells, which are believed to be responsible for recurrence and resistance to chemotherapy [26].

The identification of more reliable biomarkers of osteosarcoma in order to increase the survival odds of patients, is currently a big conundrum that needs to be addressed. Based on this aim, this study screened out the IBSP as a promising biomarker for diagnosis and prognosis prediction for osteosarcoma. The role of IBSP on the progression of osteosarcoma was investigated to provide new targets for the treatment of osteosarcoma. Further analysis of the data also revealed that higher IBSP expression was significantly associated with poor overall survival and relapse-free survival in osteosarcoma patients. This finding implies that IBSP could be a useful prognostic marker for predicting outcomes in osteosarcoma patients. It is perhaps not surprising when considering the role IBSP plays in the development of osteosarcoma.

Several biomarkers have been proposed and investigated in osteosarcoma research, but only a few have been validated and adopted in clinical practice. Ki-67 is a marker of cell proliferation and is commonly used to determine the aggressiveness and prognosis of osteosarcoma [27]. High levels of Ki-67 expression are associated with poor survival rates and increased risk of metastasis [28]. Osteopontin, an extracellular matrix protein, has been shown to promote tumor growth and invasion in several cancers, including osteosarcoma [29]. Elevated levels of osteopontin have been detected in osteosarcoma tissues and serum, and may serve as a potential biomarker for diagnosis and prognostication [30]. MicroRNAs (miRNAs), small non-coding RNA molecules, have been implicated in various biological processes, including cancer development and progression [31]. Several miRNAs have been found to be dysregulated in osteosarcoma and may have diagnostic, prognostic, and therapeutic implications [32]. Zhu et al. identified miRNA-542-5p as a critical miRNA in osteosarcoma [33]. Wang et al. found miRNA-188-5p alleviates the progression of osteosarcoma [34].

In summary, IBSP has shown promising results as a cancer biomarker, and further research is needed to fully understand its potential clinical applications in cancer diagnosis, prognosis, and targeted therapy. However, the study is limited and can be improved by exploring the related mechanism and supported by the animal experiments. While the exact mechanism and specific signaling pathways by which IBSP promotes osteosarcoma progression remains unclear, it is thought that IBSP enhances cell adhesion and migration, promoting the invasion and metastasis of cancer cells in bone tissue. Additionally, IBSP has been shown to interact with other signaling molecules, such as growth factors and extracellular matrix proteins, resulting in the activation of pro-tumorigenic pathways. These findings suggest that IBSP has a significant role in promoting tumor growth and metastasis. Overall, the development of reliable and clinically relevant biomarkers for osteosarcoma remains an active area of research. The integration of multiple biomarkers and technologies may enhance the accuracy and sensitivity of osteosarcoma diagnosis, prognosis, and treatment. For osteosarcoma patients, if early detection cannot be achieved, the treatment and nursing process of patients will face great challenges.

## CONCLUSION

Our analysis revealed that IBSP expression is significantly higher in osteosarcoma tumor tissues compared to normal bone tissues. This finding suggests that IBSP could serve as a potential biomarker for osteosarcoma, which could aid in early detection and monitoring of the disease. Further research is necessary to elucidate the specific role of IBSP in osteosarcoma progression and the design of targeted therapies to inhibit IBSP signaling in high-risk patients.

## Supplementary Materials

Supplementary Table 1
