# Supplementary material for: High expression of integrin-binding sialoprotein (IBSP) is associated with poor prognosis of osteosarcoma
Source: Aging (Albany NY). 2023 Nov 22;16(1):28–42. doi: 10.18632/aging.205235 (PMC10817378; doi:10.18632/aging.205235)
Supplement: Supplementary Table 1 [file aging-16-205235-s001.pdf]

SUPPLEMENTARY MATERIALS

Supplementary Table

Supplementary Table 1. Characteristics of patients.

Characteristics	Numbers of cases (%)
Age	
<14	15(46.88)
>14	17(53.12)
Gender	
Female	13(40.63)
Male	19(59.37)
Race	
Asian	32(100.00)
Metastasis	
met	32(100.00)
Site	
Distal	15(46.88)
Other	4(1.25)
Proximal	13(40.63)
Surgery	
Amputation	7(21.88)
Limb sparing	25(78.12)
Progression	
No	10(31.25)
Yes	22(68.75)
Vital status	
Alive	21(65.63)
Dead	11(34.37)
IBSP	
High	11(34.37)
Low	21(65.63)
